# Repeatability and response to therapy of dynamic contrast-enhanced magnetic resonance imaging biomarkers in rheumatoid arthritis in a large multicentre trial setting

**DOI:** 10.1007/s00330-017-4736-9

**Published:** 2017-01-23

**Authors:** John C. Waterton, Meilien Ho, Lars H. Nordenmark, Martin Jenkins, Julie DiCarlo, Gwenael Guillard, Caleb Roberts, Giovanni Buonaccorsi, Geoffrey J. M. Parker, Michael A. Bowes, Charles Peterfy, Herbert Kellner, Peter C. Taylor

**Affiliations:** 1 Manchester Academic Health Sciences Centre, University of Manchester, Stopford Building, Oxford Road, Manchester, M13 9PT UK; 2 Personalised Healthcare & Biomarkers, AstraZeneca, Macclesfield, UK; 3 Global Medicines Development, AstraZeneca, Macclesfield, UK; 4 Global Medicines Development, AstraZeneca, Mölndal, Sweden; 5 Global Medicines Development, AstraZeneca, Cambridge, UK; 6 Spire Sciences Inc, Boca Raton, FL USA; 7 Imorphics, Manchester, UK; 8 Bioxydyn, Manchester, UK; 9 Private Practice and Division of Rheumatology KHI Neuwittelsbach, München, Germany; 10 Kennedy Institute, University of Oxford, Oxford, UK

**Keywords:** Magnetic resonance imaging, Arthritis, rheumatoid, Fostamatinib, Adalimumab, Biological markers

## Abstract

**Objectives:**

To determine the repeatability and response to therapy of dynamic contrast-enhanced (DCE) MRI biomarkers of synovitis in the hand and wrist of rheumatoid arthritis (RA) patients, and in particular the performance of the transfer constant *K* ^*trans*^, in a multicentre trial setting.

**Methods:**

DCE-MRI and RA MRI scoring (RAMRIS) were performed with meticulous standardisation at baseline and 6 and 24 weeks in a substudy of fostamatinib monotherapy in reducing synovitis compared with placebo or adalimumab. Analysis employed statistical shape modelling to avoid biased regions-of-interest, kinetic modelling and heuristic analyses. Repeatability was also evaluated.

**Results:**

At early study termination, DCE-MRI data had been acquired from 58 patients in 19 imaging centres. *K* ^*trans*^ intra-subject coefficient of variation (N = 14) was 30%. *K* ^*trans*^ change demonstrated inferiority of fostamatinib (N = 11) relative to adalimumab (N = 10) after 6 weeks (treatment ratio = 1.92, p = 0.003), and failed to distinguish fostamatinib from placebo (N = 10, p = 0.79). RAMRIS showed superiority of fostamatinib relative to placebo at 6 weeks (p = 0.023), and did not distinguish fostamatinib from adalimumab at either 6 (p = 0.175) or 24 (p = 0.230) weeks.

**Conclusion:**

This demonstrated repeatability of *K* ^*trans*^ and its ability to distinguish treatment groups show that DCE-MRI biomarkers are suitable for use in multicentre RA trials.

***Key Points*:**

*• DCE-MRI biomarkers are feasible in large multicentre studies of joint inflammation.*

*• DCE-MRI K* ^*trans*^ *showed fostamatinib inferior to adalimumab after 6 weeks.*

*• K* ^*trans*^ *repeatability coefficient of variation was 30*% *multicentre.*

**Electronic supplementary material:**

The online version of this article (doi:10.1007/s00330-017-4736-9) contains supplementary material, which is available to authorized users.

## Introduction

MRI with gadolinium-based contrast agents (Gd-CAs) provides biomarkers dependent on perfusion, vascular volume, capillary endothelial permeability and interstitial volume, all of which increase in inflammation. MRI is widely available, sensitive, a low risk to patients and amenable to quantitation. OMERACT (Outcome Measures in Rheumatology) RAMRIS (Rheumatoid Arthritis MRI scoring) [1, 2] synovitis score is well established [3], but, as an ordinal variable, is theoretically less sensitive than a continuous variable [4] as a biomarker. Also, RAMRIS reports amount (an ‘extensive’ variable), but not severity (an ‘intensive’ variable), of synovitis, and cannot distinguish the importance of extent versus intensity of inflammation in RA, which is currently unknown.

Dynamic contrast enhanced (DCE) MRI [5] characterises regional uptake and washout of Gd-CA. It has been extensively used in oncology [6] and other diseases, and in RA provides biomarkers of synovial inflammation [7]. Despite over 60 DCE-MRI RA studies (over 1,000 patients) in PubMed, DCE-MRI RA studies until recently [8] were performed only in single expert centres, or occasionally [9] in two centres with identical equipment. RAMRIS, however, is routinely employed in large multicentre studies using different vendors’ MRI equipment [3]. A likely reason for failure to exploit DCE-MRI in multicentre RA studies is that the heuristic variables commonly used to characterise synovial Gd-CA uptake curves are inherently scanner-dependent, and therefore unlikely to provide biomarker values comparable between centres and studies. Also, as with any intensive variable, DCE-MRI biomarkers depend on how their region-of-interest (ROI) is defined, and because of variations in patient positioning and other technical factors, it is difficult to ensure that ROIs correspond between time points and subjects.

We reasoned that with rigorous site qualification and scanner monitoring, objective definition of ROIs by statistical shape modelling and robustly quantified compartmental modelling, we could reliably measure DCE-MRI biomarkers even in a large multicentre study using a variety of MRI equipment in centres with little or no previous quantitative DCE-MRI experience.

Here we present multicentre DCE-MRI, repeatability and response to treatment, in a study [10] of fostamatinib [11].

## Methods (see Supplementary Material for detail)

### Patients and treatment

The MRI substudy to OSKIRA-4 (Oral SYK Inhibition in Rheumatoid Arthritis) [10] (ClinicalTrials.gov Identifier: NCT02092961) was a Phase IIB, multicentre, randomised, double-blind, placebo-controlled, parallel-group study of efficacy and safety of fostamatinib disodium (a spleen tyrosine kinase inhibitor) monotherapy, compared with placebo or adalimumab monotherapy in patients with active RA. The primary substudy objective was to assess the efficacy of fostamatinib in reducing joint synovial disease activity as measured by change from baseline to week 6 (vs. placebo) in OMERACT RAMRIS synovitis score. Exploratory objectives included assessment of efficacy of fostamatinib in reducing joint synovial disease activity as measured by change from baseline to week 6 (vs. placebo and adalimumab) and week 24 (vs. adalimumab) in certain DCE-MRI biomarkers including *K* ^*trans*^.

The findings of the full OSKIRA-4 clinical trial are reported elsewhere [10]. All patients gave written informed consent. Patients were DMARD-naïve, intolerant to DMARDs or had had inadequate response to maximally two DMARDs. Patients were randomised to one of three treatments: fostamatinib (100 mg bid for 24 weeks plus placebo subcutaneous injection every 2 weeks); adalimumab (40 mg subcutaneous injection every 2 weeks for 24 weeks, plus placebo to fostamatinib bid); placebo bid for 6 weeks followed by switch to 100 mg fostamatinib bid up to week 24, plus placebo subcutaneous injection every 2 weeks.

The more clinically active hand and wrist was imaged at screening, week 6 and week 24 using 3.0 T or 1.5 T whole-body MRI, with knee coils to allow simultaneous scanning of MCP and wrist joints. An acrylic frame ensured reproducible hand/wrist positioning. Some patients provided an additional baseline scan before the first dose of randomised treatment. All randomised patients were to have contrast-enhanced MRI (CE-MRI) assessments at the scheduled time points. Where participating sites could demonstrate acceptable DCE-MRI performance, this more technically demanding acquisition (subject of this report) was also performed. Approximately 20 patients in each dosing regimen were planned to have DCE-MRI evaluable at baseline, week 6 and week 24. On 4 June 2013, AstraZeneca announced results from Phase III trials of fostamatinib, and its decision not to proceed with regulatory filings, following which this study was terminated early.

### DCE-MRI biomarkers

**Fig. 1:**
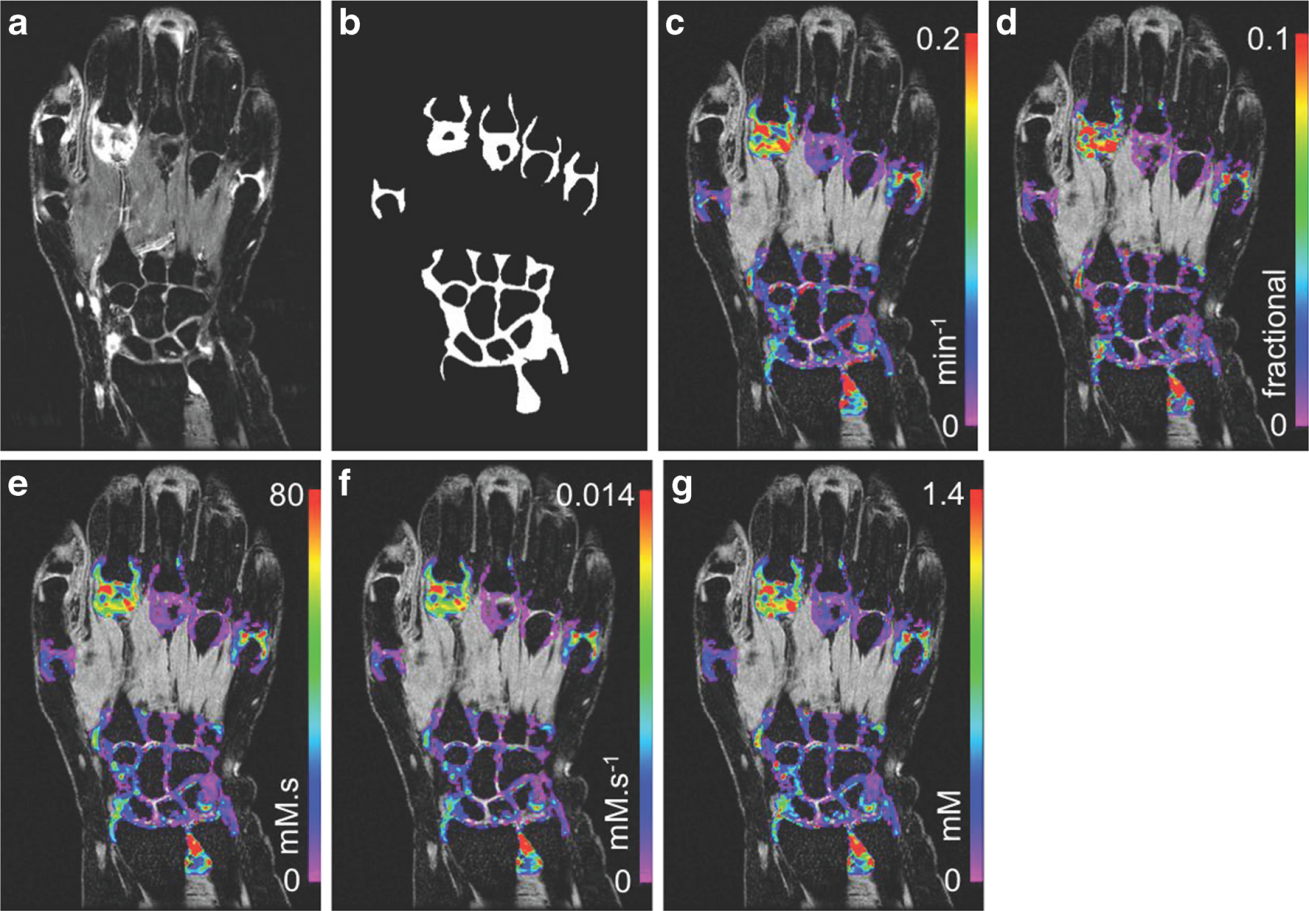
Post-contrast high resolution *T*_*1*_-weighted spoiled gradient-recalled echo (SPGR) image with fat saturation shows predominant disease in the second metacarpal joint (MCP) and isolated areas of disease in MCP-5, the distal radio-ulnar and radio-carpal joints (**a**). Segmented joint voxel masks were produced for each joint and used in the DCE-MRI analysis (**b**). Pre-contrast images with DCE-MRI parameterisation overlays for: *K* ^*trans*^ (**c**), *v*_*p*_ (**d**), *IAUC*_*120*_ (**e**), *IRE* (**f**) and *ME* (**g**)

Pre-specified DCE-MRI biomarkers (Fig. 1c–g), in priority order, were:

- *K* ^*trans*^/min^-1^: volume transfer constant for Gd-CA between blood plasma and extravascular extracellular space from extended Tofts [5] compartmental model.
- *IRE/* mM.s^-1^: initial rate (gradient) of enhancement following Gd-CA over 60 s post-arrival in tissue.
- *IAUC*_*60*_, *IAUC*_*120*_/mM.s: initial area under Gd-CA concentration curve over 60 s or 120 s post-arrival in tissue.
- *VEP*/mL: volume of enhancing pannus [12].
- *ME*/mM: maximum enhancement of Gd-CA concentration curve during DCE-MRI series.
- *v*_*e*_, *v*_*p*_: volumes respectively of extravascular extracellular space, and blood plasma volume, per unit volume tissue (dimensionless).

Each biomarker was measured voxelwise over a ROI defined objectively using statistical shape modelling.

### Statistical analysis

Analyses followed a predetermined statistical analysis plan, finalised and signed prior to locking the database and unblinding. Fostamatinib was compared pairwise with placebo (week 6) and adalimumab (weeks 6 and 24). Endpoints were tested at a two-sided significance level of 10%. Double baseline DCE-MRI repeatability was assessed using a mixed model of the two baseline results only.

## Results

No new safety findings inconsistent with the known profiles of adalimumab, fostamatinib or any of the contrast agents were reported in the substudy. Prone MRI, with arm over head, may be uncomfortable for patients, but most tolerated the procedure well, providing images without unacceptable motion artefact (Fig. 1). Although the DCE-MRI protocol is not longer than the CE-MRI protocol, for DCE-MRI patients must remain immobile for the entire 26- to 28-min scan. We did not prospectively seek to evaluate relative tolerability of DCE-MRI and CE-MRI; informal records, however, indicate that of 64 who had both, two were unable to remain still during the dynamic scans because of discomfort, resulting in excessive motion artefact, and transferred to the CE-MRI-only cohort after baseline. In addition, four whose DCE-MRI failed quality control at baseline declined repeat scans because of discomfort and unwillingness to repeat MRI so quickly. No other patient withdrew for reasons connected with MRI.

Fourteen patients from six centres provided two valid DCE-MRI datasets and RAMRIS scores at baseline allowing repeatability to be determined (Table 1). Demography was generally balanced across treatment arms (Table S1). Because of early study termination, fewer data were accrued than planned: 58 patients (from 19 centres) provided technically valid DCE-MRI (Fig. S6); 45 were randomised and treated; 31 (52% of target) provided DCE-MRI and RAMRIS scores at week 6, and 19 (32% of target) at week 24 (Table 2). At week 6, *K* ^*trans*^ was 66% of baseline (geometric mean, N = 10) with adalimumab (range 27–154%); 104% of baseline (N = 11) (66–240%) with fostamatinib; and 124% of baseline (N = 10) (66–517%) with placebo (Fig. S2).

**Table 1:** Repeatability and range, averaging over diseased and non-diseased joints

MR biomarker	Patients with two baseline scans			Treated patients at baseline		
	N for repeatability	Repeatability (intra-subject CoV %)	Variability (inter-subject CoV %)	N for range	Geometric mean	Range
*K* ^*trans*^ (min^-1^)	14	30.0%	59.3%	45	0.069	0.025–0.273
*IRE* (mM.s^-1^)	14	29.5%	51.3%	45	0.003	0.001–0.013
*IAUC*_*60*_ (mM.s)	14	31.4%	58.3%	45	7.69	1.92–28.17
*IAUC*_*120*_ (mM.s)	14	29.3%	55.7%	45	18.23	5.58–70.27
*VEP* (mL)	15	22.0%	41.3%	45	1.06	0.20–2.48*
*ME* (mM)	14	27.5%	52.4%	45	0.32	0.11–1.31
*v*_*e*_ (%)	0	n/a	n/a	0	n/a	n/a
*v*_*p*_ (%)	14	53.4%	60.1%	45	1.2	0.3–4.5
RAMRIS synovitis score**	13	1.30 (SD)	6.20 (SD)	31	6.95 (arithmetic mean)	0.0–21.8

*VEP excludes non-enhancing pannus. The range for total (non-enhancing plus enhancing) pannus was 3.29-8.30 ml

** *VEP* and the DCE-MRI endpoints are log-normally distributed but RAMRIS synovitis score is not. Therefore geometric mean and intra-subject CoV were calculated for *VEP* and the DCE-MRI endpoints, while arithmetic mean and intra-subject SD were calculated for RAMRIS synovitis score. The intra-class correlation coefficients were respectively 0.958 for RAMRIS synovitis score and 0.777 for *K* ^*trans*^. RAMRIS synovitis score is based on an ordinal scale (0–24) so there is a restriction on how many values the synovitis score can actually take, therefore increasing the chance of a repeatable result. Given this, a direct comparison in repeatability with *K* ^*trans*^ is difficult

*CoV* coefficient of variation, *DCE-MRI* dynamic contrast-enhanced MRI, *IAUC*_*60*_ *IAUC*_*120*_/mM.s initial area under the Gd-CA concentration curve over 60 s or 120 s post-arrival in tissue, *IRE/* mM.s^-1^ initial rate (gradient) of enhancement following Gd-CA over 60 s post-arrival in tissue, *K* ^*trans*^/min^-1^ volume transfer constant for Gd-CA between blood plasma and extravascular extracellular space, *ME*/mM maximum enhancement of Gd-CA concentration curve during DCE-MRI series, *RAMRIS* RA MRI score, *SD* standard deviation, *T*_*1*_ /s longitudinal relaxation time, *v*_*e*_ volume of extravascular extracellular space per unit volume tissue, *v*_*p*_ volume of blood plasma volume per unit volume tissue, *VEP*/ml volume of enhancing pannus

**Table 2:** Change from baseline of the synovial MRI biomarkers in response to intervention

Biomarker	Fostamatinib (N = 11) vs. placebo (N = 10) at 6 weeks		Fostamatinib (N = 11) vs. adalimumab (N = 10) at 6 weeks		Fostamatinib (N = 6) vs. adalimumab (N = 5) at 24 weeks	
	Treatment ratio* (tr) or difference (td) (90% CI)	Two-sided p-value	Treatment ratio* (tr) or difference (td) (90% CI)	Two-sided p-value	Treatment ratio* (tr) or difference (td) (90% CI)	Two-sided p-value
*K* ^*trans*^	tr = 0.95 (0.68–1.33)	0.794	tr = 1.92 (1.36–2.72)	0.003	tr = 1.59 (0.95–2.68)	0.137
*IRE*	tr = 0.86 (0.62–1.18)	0.417	tr = 1.55 (1.12–2.15)	0.031	tr = 1.60 (1.06–2.42)	0.064
*IAUC*_*60*_	tr = 0.91 (0.66–1.24)	0.603	tr = 1.67 (1.21–2.30)	0.012	tr = 1.57 (0.97–2.54)	0.120
*IAUC*_*120*_	tr = 0.88 (0.65–1.19)	0.478	tr = 1.67 (1.22–2.28)	0.010	tr = 1.60 (0.98–2.61)	0.116
*VEP*	tr = 0.85 (0.66–1.08)	0.130	tr = 0.77 (0.60–1.00)	0.053	tr = 1.23 (0.62–2.11)	0.508
*ME*	tr = 0.95 (0.70–1.28)	0.756	tr = 1.64 (1.20–2.25)	0.012	tr = 1.57 (1.05–2.36)	0.070
*v*_*e*_	n/a	n/a	n/a	n/a	n/a	n/a
*v*_*p*_	tr = 0.90 (0.60–1.29)	0.610	tr = 1.75 (1.21–2.54)	0.016	tr = 1.75 (1.21–2.54)	0.065
RAMRIS synovitis score	td = -2.00 (-3.25–-0.75)	0.023	td = -1.50 (-2.50–0.00)	0.175	td = 2.00 (-0.50–5.00)	0.230
DAS-28 CRP	td = 0.65 (-0.11–1.41)	0.155	td = -0.13 (-0.89–0.64)	0.780	td = -1.48 (-3.43–0.47)	0.200

Based on the response at 6 weeks in the adalimumab group, the standardised response mean for *K* ^*trans*^ was -0.64

*DAS-28 CRP* Disease Activity Score 28 based on C-reactive protein

*tr is used for *VEP* and the DCE-MRI endpoints as they are log-normally distributed but td for the RAMRIS synovitis score and DAS-28 CRP which are not:

tr <1 or td < 0 indicate an effect in favour of fostamatinib

## Discussion

MRI biomarkers [13] pose different challenges to soluble biomarkers. Biomarker quality and validity depends on operation of an MRI device not primarily designed for quantitative work, perhaps in a manner unfamiliar to users in trial sites. Encouraging measures of repeatability and response to therapy in small studies in single expert centres may not translate to real-world multicentre trials. It is therefore necessary to evaluate [14] these biomarkers specifically in the multicentre setting.

Previously, various MRI biomarkers have been derived from Gd-CA-enhanced images. The rationale for selecting our preferred DCE-MRI biomarker, *K* ^*trans*^, and our exploratory biomarkers, is described in detail in the Supplementary Material. While many previous DCE-MRI studies in RA used variants of the heuristic parameters *IRE* and *ME*, in this work we followed international standardisation projects and guidelines [15] in employing *K* ^*trans*^. Unlike the case with CT or nuclear medicine, in DCE-MRI the signal intensity has a non-linear relationship to Gd-CA concentration [16] which depends in a complex way on baseline *T*_*1*_, *B*_*1*_ heterogeneity, flow artefacts, pulse sequence parameters and post-processing. Metrics dependent on the native signal intensities necessarily incorporate these dependencies, and while there should little effect on repeatability when patients are imaged in the same scanner, they make values difficult to compare between scanners (or even between upgrades of the same scanner). Thus while the MR signal intensity-based heuristic biomarkers can exhibit good repeatability single-centre [17] they were inappropriate for the present study.

For our preferred DCE-MRI biomarker, *K* ^*trans*^, the multicentre intra-subject repeatability coefficient of variation (CoV) of 30% was similar to a previous single-centre RA report [18] but worse than typically seen in oncology studies [6] where repeatability CoV of 15% is more typical. This likely reflects our choice to average over all joints including those with little (or no) synovitis. At week 6 *K* ^*trans*^ clearly distinguished the inferior effect of fostamatinib from adalimumab on synovial inflammation, but failed to distinguish fostamatinib from placebo. This is interpreted as an early effect of adalimumab, but not fostamatinib, on synovial capillary blood flow and/or capillary endothelial permeability. The main OSKIRA-4 [10] study (N = 279) concluded fostamatinib at the two higher dose regimens was more efficacious than placebo at week 6 but less than adalimumab at week 24 in terms of RA signs and symptoms. In this substudy, RAMRIS findings are consistent with the main study at week 6, but the number of substudy patients at week 24 was too small to draw firm conclusions. Heuristic biomarkers *IAUC*_*120*_, *IAUC*_*60*_, *IRE* and *ME* exhibited similar repeatability to *K* ^*trans*^, and similarly distinguished fostamatinib from adalimumab. We derived *IRE* and *ME* in absolute not arbitrary (machine-dependent) units, which probably reduced scanner-related variation from what would be expected from signal intensity-based heuristics. *v*_*p*_ is challenging to measure, as blood plasma constitutes only a small volume fraction of the synovitis (here around 1.5%) but it did distinguish fostamatinib from adalimumab despite worse repeatability than the other biomarkers.

Given this variability in *K* ^*trans*^, a future parallel group DCE-MRI study of 20 patients per arm would give 80% power to detect a treatment ratio of 1.67 at a two-sided significance level of 10%.

Due to early study termination, comparisons between groups should be interpreted with caution. All biomarkers were exploratory without correction for multiple comparisons. The study was not designed to compare adalimumab with placebo, nor to compare DCE-MRI with RAMRIS, nor to test whether early MRI changes forecast clinical outcome. However, our findings from this truncated study do demonstrate that DCE-MRI biomarkers are feasible and sensitive in a large multicentre trial setting.

## Electronic supplementary material

Below is the link to the electronic supplementary material.

ESM 1 (DOCX 4896 kb)

## Abbreviations and acronyms

bid: Twice daily
CE-MRI: Contrast-enhanced MRI
CoV: Coefficient of variation
DAS-28 CRP: Disease Activity Score calculated from 28 joints and using C-reactive protein
DCE-MRI: Dynamic contrast-enhanced MRI
DMARD: Disease-modifying anti-rheumatic drug
Gd-CA: Gadolinium-based contrast agent
*IAUC*_*60*_*IAUC*_*120*_/mM.s: Initial area under the Gd-CA concentration curve over 60 s or 120 s post-arrival in tissue
*IRE/* mM.s^-1^: Initial rate (gradient) of enhancement following Gd-CA over 60 s post-arrival in tissue
*K*^*trans*^/min^-1^: Volume transfer constant for Gd-CA between blood plasma and extravascular extracellular space
MCP: Metacarpophalangeal
*ME*/mM: Maximum enhancement of Gd-CA concentration curve during DCE-MRI series
MRI: Magnetic resonance imaging
OMERACT: Outcome measures in rheumatology initiative
OSKIRA: Oral SYK inhibition in RA
QC: Quality control
RA: Rheumatoid arthritis
RAMRIS: RA MRI score
ROI: Region of interest
SD: Standard deviation
SPGR: Spoiled gradient-recalled echo
SYK: Spleen tyrosine kinase
*T*_*1*_ /s: Longitudinal relaxation time
td: Treatment difference
tr: Treatment ratio
*v*_*e*_: Volume of extravascular extracellular space per unit volume tissue
*v*_*p*_: Volume of blood plasma volume per unit volume tissue
*VEP*/ml: Volume of enhancing pannus
