# Supplementary material for: Repeatability and response to therapy of dynamic contrast-enhanced magnetic resonance imaging biomarkers in rheumatoid arthritis in a large multicentre trial setting
Source: Eur Radiol. 2017 Jan 23;27(9):3662–8. doi: 10.1007/s00330-017-4736-9 (PMC5544811; doi:10.1007/s00330-017-4736-9)
Supplement: Supplementary file 1 — (DOCX 4896 kb) [file 330_2017_4736_MOESM1_ESM.docx]

**Repeatability and Response to Therapy of**

**Dynamic Contrast-Enhanced Magnetic Resonance Imaging Biomarkers**

**in Rheumatoid Arthritis in a Large Multicentre Trial Setting.**

# Supplementary material.

##

# Demography, baseline disease characteristics and patient disposition.

The MRI sub-study to OSKIRA-4 (ClinicalTrials.gov Identifier for the sub-study: NCT02092961)[10] was a Phase IIB, multi-centre, randomised, double-blind, placebo-controlled, parallel group study of the efficacy and safety of fostamatinib disodium monotherapy compared with placebo or adalimumab monotherapy in patients with active RA, with the primary substudy objective to assess the efficacy of fostamatinib in reducing joint synovial disease activity as measured by change from baseline to week 6 (*versus* placebo) in OMERACT RAMRIS synovitis score. Exploratory objectives included assessment of the efficacy of fostamatinib in reducing joint synovial disease activity as measured by change from baseline to week 6 (*versus* placebo and adalimumab) and week 24 (*versus* adalimumab) in certain DCE-MRI parameters including *K^trans^* (/min^-1^). All patients gave written informed consent. Eligible patients included those who were not currently receiving disease-modifying anti‑rheumatic drugs (DMARDs) (DMARD naïve, intolerant to DMARDs or had had an inadequate response to at most two DMARDs), and who had swelling at clinical assessment, and the presence of MRI synovitis in least one MCP or wrist joint. Patients must not have used DMARDs within 6 weeks prior to screening in order to be eligible to participate in the study. Also ineligible were patients for whom MRI or Gd-based contrast agents were contra-indicated. Patients were randomised to one of three treatments: F (fostamatinib 100mg *bid* for 24 weeks plus placebo subcutaneous injection every 2 weeks); A (adalimumab 40 mg by subcutaneous injection every 2 weeks for 24 weeks, plus placebo to fostamatinib twice daily); or P (placebo *bid* for 6 weeks followed by switch to 100 mg fostamatinib *bid* up to week 24, plus placebo subcutaneous injection every 2 weeks). A double-dummy blinding technique was used to ensure neither patient nor investigator would know which treatment the patient was receiving. Since the exact placebo matching adalimumab (Humira, AbbVie) subcutaneous injection was not available, each site was required to appoint an unblinded administrator for the subcutaneous injections every 2 weeks, who was independent of the rest of the on-site study team.

Enrolment of the required number of patients into the main OSKIRA-4 study was completed in March 2012. However, at this stage only two patients had been recruited to the sub study, which is therefore analysed separately, and no combined analyses of the main study and sub-study were performed. MRI was performed at screening (6-14 days prior to first dose), and at week 6 (+5/-2 days) and week 24 (±5 days). In addition, for some patients, where appropriate optional patient consent was given, an additional baseline scan was performed at least 2 days prior to the first dose of randomised treatment, with at least 3 days separating the two baseline scans, to establish the repeatability of the DCE-MRI biomarkers in this multicentre setting. All randomized patients were to have Contrast–Enhanced MRI (CE-MRI) assessments performed at the scheduled time points. Where participating sites could demonstrate acceptable DCE-MRI performance, both CE and DCE-MRI parameters were obtained.

The findings of the main OSKIRA-4 study are reported elsewhere[10]. In the OSKIRA-4 MRI substudy, 198 patients were enrolled, of whom 97 were randomised (34 F, 33 A, 30 P) and 101 excluded. Thirty-two randomised subjects from the sub-study had evaluable DCE-MRI data from both baseline and Week 6 and/or 24, this analysis set being the focus of this manuscript. Demography was generally balanced across treatment arms (Supplementary table 1) considering the relatively small numbers.

**Supplementary table 1. Demographic and baseline RA disease characteristics**

|  | | Fostamatinib 100 mg *bid* n=11 | Adalimumab 40mg (2 weekly) n=10 | Placebo (6 weeks) then fostamatinib 100mg *bid* n=10 |
| --- | --- | --- | --- | --- |
| Age (yrs) | Median, range | 58 (33, 74) | 55 (30, 70) | 47 (23, 74) |
| Female | n (%) | 9(82) | 8(80) | 7(70) |
| Swollen joint count | Mean (SD) | 10 (4) | 13 (7) | 11 (4) |
| Tender joint count | Mean (SD) | 16 (5) | 15 (7) | 14 (5) |
| HAQ-DI score | Mean (SD) | 1.7 (0.61) | 1.2 (0.58) | 1.5 (0.65) |
| CRP (mg.L^-1^) | Median (range) | 14 (1, 60) | 10(1, 72) | 17 (1, 79) |
| ESR (mm.h^-1^) | Median (range) | 38 (30, 128) | 46 (28, 68) | 38 (23, 107) |
| DAS28 score | Mean (SD) | 5.96 (0.70) | 5.63 (1.26) | 5.60 (0.72) |
| Years since RA first diagnosed | Median (range) | 1.4 (0.1, 3.7) | 0.3 (0.1, 3.1) | 1.0 (0.1, 5.0) |
| Rheumatoid factor positive* | n (%) | 10 (91) | 10 (100) | 9 (90) |
| Radiological erosions present | n (%) | 3 (27) | 2 (20) | 4 (40) |
| DMARD-naïve | n (%) | 5 (45) | 7 (70) | 6 (60) |
| DMARD-IR / Intolerant | n (%) | 6 (55) | 3 (30) | 4 (40) |

*These values are from the medical history. In the tests conducted at baseline the numbers positive were respectively 9, 8, 9.

# Rationale for selection of imaging biomarkers.

The introduction of a number of effective new therapies for RA in the past decade, while very welcome, creates challenges for the rheumatologist and drug developer, as it becomes more difficult to design feasible studies to compare investigational with established agents, or to identify which RA populations obtain the most benefit from any particular treatment. This is particularly so when employing outcome measures comprising composite scores of disease activity where the clinical assessment components are binary measures, and an assessed joint is evaluated as either being swollen or non-swollen, tender or non-tender. Such evaluations risk failure to detect more subtle levels of synovitis, and are relatively insensitive to detect change on therapeutic intervention. Imaging biomarkers have the potential to improve sensitivity to change by providing quantitative assessments of joint inflammation. Regulatory agencies recognise[15,16] that slowing of radiographic progression may offer a surrogate for long-term preservation of functional status. However, the agencies note that X-radiography of joint destruction is increasingly challenging because of slow progression if an active comparator is used, while MRI assessment of structural change is not considered sufficiently validated as an outcome measure for pivotal phase III clinical trials. In early drug development, imaging biomarkers of inflammation are attractive because they exhibit much faster and larger changes than structural biomarkers, making quantitative MR imaging biomarkers attractive for use in clinical trials. Subclinical MRI-detected synovitis and osteitis occur frequently in RA patients even when they are in clinical remission and may contribute to structural progression in these patients[17], although the relationship between synovial inflammation, osteitis, pain, erosion, and disability is complex[18]. Ideally, such biomarkers would be highly repeatable, reproducible between centres, and sensitive to change, to permit exploration of different doses, combinations, and patient groups in small short-term phase 2 studies, whose conclusions would improve the design of subsequent conventional pivotal phase 3 studies.

The OMERACT RAMRIS (Rheumatoid Arthritis MRI scoring system)[1,2] synovitis score has previously been widely used with considerable success[3] and has previously[19] been able in an 18-centre study to detect treatment-induced improvement in synovitis within as little as 2 weeks in a 32-patient cohort. Both adalimumab[20,21] and fostamatinib[11] have reported beneficial effects on RAMRIS-determined synovitis. Eight previous multicentre randomised controlled trials have shown reductions in RAMRIS synovitis score over 2-26 weeks with group sizes of 29-159 per arm[3,8]. As a measurement of the extent of synovitis, RAMRIS synovitis score is repeatable and reproducible between centres, but its scale includes only 24 increments In general[4], an ordinal variable such as RAMRIS synovitis score may often have less discriminatory ability to pick up small changes than a continuous variable representing similar underlying biology, although RAMRIS has shown discriminative power at only 2 weeks in multi-centre randomised controlled trials with 30 or fewer patients per arm[8]. DCE-MRI biomarkers, however, can measure both the extent and intensity of synovitis as continuous variables, and may therefore be more sensitive to change than is RAMRIS synovitis, but there is little evidence to date that DCE-MRI biomarkers are reproducible between centres. Several small single centre studies (in one case two expert centres with identical equipment) have shown treatment-associated reductions in the heuristic parameter *IRE*, for example by 31% in 10 patients following 26 weeks rituximab[22], by 35% in 15 patients following 18 weeks abatacept[23], by 32% in 19 patients following 14 weeks methotrexate and infliximab[24], by 33% in 18 patients 1 day following intra-articular methylprednisolone in the knee[25], by 29% in 13 patients following 1 week anti-TNF therapy[26], and by 10% in 17 patients following 17 weeks leflunomide[9]. *IRE*, in its common implementation, appears unsuitable for general multicentre use because it is based on MRI signal intensity changes, which are platform-dependent, leading to measurements in arbitrary-units.s^-1^. Compartmental modelling has been less-used, however a reduction in *K^trans^* of 31% in 13 patients single-centre following 1 week of anti-TNF therapy has been reported[26].

In this study the prespecified DCE-MRI biomarkers, each measured over a region-of-interest defined objectively through 3D statistical shape modelling described below, were (in priority order):

*K^trans^*: Volume transfer constant between blood plasma and extravascular extracellular space (also referred to as the transfer coefficient of contrast agent across the capillary membrane). *K^trans^* was measured voxelwise using the extended Tofts[5] compartmental model and has dimensions min^-1^. This “extended” model allows voxels to include non-negligible plasma volume fraction. A population arterial input function (AIF) was used for all patients due to the difficulty in reliably measuring AIFs in the vessels supplying the hand and wrist[27]. All input functions were corrected for contrast agent relaxivity and patient haematocrit[28]. This model provides estimates of the volume transfer coefficient of the contrast agent *K*^trans^, the leakage volume *v_e_* and the blood plasma volume *v_p_* to be extracted voxelwise within the regions of interest.

*IRE*: Initial rate (gradient) of enhancement of the contrast agent concentration curve over the first 60 s post contrast agent arrival in tissue. Many previous implementations derived this heuristic parameter this from the voxelwise SI(t) (Signal Intensity – time) curve, where SI has dimensions of arbitrary (machine-specific) units. Such an implementation would be very difficult to standardise validly across scanners, field strengths, image acquisition protocols, and contrast agents. In this study, therefore, we elected to use the measured voxelwise longitudinal relaxation times (*T_1_* /s), in combination with the known relaxivity of the respective contrast agent, to fit the extended Tofts model and derive a noise-free uptake curve in units of contrast agent concentration. *IRE* was subsequently estimated from the average gradient of this concentration time curve between 0 and 60 s; the use of the fitted curve reduced the impact of signal noise and variable temporal resolution of acquisition on the *IRE* definition. This provided voxelwise *IRE* with dimensions mM.s^-1^, rather than arbitrary-units.s^-1^.

*IAUC_60_* and *IAUC_120_*: Initial area under the contrast agent concentration curve (obtained from the fitted, noise-free, extended Tofts curve) over the first 60 or 120 s respectively post contrast agent arrival in tissue, measured voxelwise with dimensions mM.s. *IAUC* has sometimes been preferred[29] to *K^trans^* because *IAUC* does not employ compartmental modelling so is not vulnerable to fitting failures. *IAUC_120_* has sometimes been preferred to *IAUC_60_*, particularly with older instruments that cannot achieve good time resolution in dynamic acquisition.

*VEP*: Volume of enhancing pannus[12] in mL. The workflow to identify enhancing voxels is further described in the “Region-of-interest Definition” section in this supplement.

*ME*: Maximum enhancement of the contrast agent concentration curve during DCE-MRI series. As for *IRE*, the heuristic parameter *ME* was obtained voxelwise (from the fitted, noise-free, extended Tofts curve) not as previously in arbitrary units, but in absolute units of mM.

*v_e_* and *v_p_*: Volumes respectively of extravascular extracellular space, and blood plasma volume, per unit volume *of* tissue accessible to contrast agent obtained from the extended Tofts model (both dimensionless).

Some investigators advocate *K^trans^* and *v*_p_ as biomarkers as they admit more direct physiologic interpretation than *IAUC, IRE* or *ME*.

In addition to the DCE-MRI biomarkers, OMERACT RAMRIS synovitis score was also obtained. The full RAMRIS findings (synovitis, osteitis, erosions) from all the patients in the MR substudy, including those who did not provide DCE-MRI data, will be published elsewhere.

The DCE-MRI biomarkers (*i.e.* characterising the uptake curve) *K^trans^*, *IRE*, *ME*, *IAUC*, *v_p_* and *v_e_* are “intensive” parameters, i.e. they attempt to measure the “intensity” of inflammation rather than its spatial extent. In comparison, the two CE-MRI biomarkers (*i.e.* pre- *vs*. post-Gd-CA), *VEP* and RAMRIS synovitis score, are “extensive” parameters, *i.e.* they attempt to measure the spatial extent of inflammation. *K^trans^*, reflecting the intensity of the synovial inflammation, may provide different insights than do *VEP* or RAMRIS synovitis score, which reflect its extent (although extent and intensity may covary). Both CE-MRI and DCE-MRI incur a similar burden to patients (Gd-CA injection and time in magnet), as in our implementation DCE-MRI was measured in the time between the pre- and post-Gd-CA scans required for CE-MRI. (This however curtailed the dynamic acquisition before some voxels’ Gd-CA concentration had reached its maximum as shown in Supplementary figure 5, and in consequence the fitting algorithm could not provide valid values of *v_e_*). Since some trial sites may be unfamiliar with quantitative DCE-MRI, additional efforts[30] are needed to qualify and standardise sites.

# DCE-MRI site qualification

DCE-MRI required additional set-up, training and QA, beyond that necessary for a site to provide RAMRIS biomarkers. A total of 32 imaging centres, in the USA (N=14), Europe (N=15), and Africa (N=2), were trained and set-up specifically to participate in the DCE-MRI study, following a standardised training procedure. Three centres withdrew during the site qualification process due to insufficient hardware capabilities that made them ineligible to participate in the DCE-MRI protocol. 29 centres were successfully qualified representing all three major vendors (GEHC, Philips, and Siemens) with 1.5T and 3T equipment; however one centre was discontinued after two subject baseline scans due to insufficient image quality (an alternative imaging facility was found and subjects were transferred).

Centres were only considered for inclusion if they could provide MRI at 1.5T or 3T, from one of the above three vendors, of current or recent design, together with a dedicated wrist or knee coil that provided coverage of the wrist and hand, and power injector capable of administering contrast agent at the specified rate and volumes (Supplementary table 2). In order to standardise the imaging sequences and procedures across a large number of centres, radiographers/technologists were trained at each centre, and specified data acquired with a centrally-provided and standardised *T_1_*-calibration phantom. Patients were not entered into the study until adequate phantom data had been received at the DCE-MRI analysis centre.

All sites were provided with a phantom based on the Eurospin[31] Test Object TO5 (Diagnostic Sonar, Livingstone, Scotland). Five gel tubes were included with known *T_1_* values (*T_1_* range 210 – 1470 ms at 1.5T and 21 °C). Sites were trained in using the scanning protocol and then required to perform the acquisition outlined in Supplementary table 3 (but without contrast agent administration) on the phantom after the training visit. Thermometers were also provided to the sites, and site personnel recorded the scanner room temperature when the phantom was scanned. Data and temperature were uploaded to the central analysis site (Bioxydyn Limited, Manchester, UK), where the calculated values of *T_1_* were checked against reference values. Successful generation of valid *T_1_* values qualified the site to continue in the study.

**Supplementary table 2: MRI scanners and coils used in this study, by manufacturer and field strength (T).**

| ***Make*** | GE Healthcare (9) | Siemens (6) | Philips (4) | |
| --- | --- | --- | --- | --- |
| ***Field strength*** | 1.5T | 1.5T | 1.5T | 3.0T |
| ***Model*** | Signa HDxt (7) | Magnetom Avanto (3) | Achieva (2) | Achieva (2) |
|  | Signa Excite (1) | Magnetom Aera (2) |  |  |
|  | Optima MR360 (1) | Magnetom Symphony (1) |  |  |
| ***Coil*** | Invivo quadrature array knee (4) | Circularized polarity extremity (3) | 8-channel knee (2) | Small extremity 8-channel (1) |
|  | Invivo 8-channel knee (4) | 15-channel transmit/receive knee (2) |  | 8-channel (1) |
|  | Medrad small extremity knee (1) | Invivo quadrature array knee (1) |  |  |

Number in parentheses represents the number of centres using that hardware. The table includes only the 20 imaging centres who successfully submitted at least one analysable DCE-MRI data set to the study. In some cases a single imaging centre was used by more than one investigator.

# CE-MRI and DCE-MRI acquisition

All patients had MRI of the more clinically active hand/wrist using a 3.0T or 1.5T whole-body scanner at one of 20 clinical centres. An acrylic frame (‘M-frame’[32], Spire sciences, Boca Raton, FL) was used to ensure fixed, reproducible positioning of the hand and wrist joints on serial MRI examinations. The scanning protocol was standardized across all sites and on all scanning platforms to help minimize variation between sites. Scans were performed with the patients in the prone position, with the hand/wrist above the head in the centre of the magnet bore, and used volume knee coils that enabled the wrist, MCP and proximal interphalangeal (PIP) joints to be included within a single field of view (FOV), except for a single site, which scanned patients in the supine position and used an extremity coil that also allowed full coverage of the hand/wrist (Supplementary Table 2).

The scanning protocol was standardized across all sites and on all scanning platforms to help minimize variation between sites. Supplementary Table 3 shows typical scanning parameters for the Philips, Siemens and GE scanners used in this study. Of the 28 imaging centres trained and set-up to participate in the study, 19 centres had successfully acquired and submitted analysable DCE-MRI data by the time the study was terminated, as shown in Supplementary Figure 6. All images were centrally evaluated for adequate image quality before inclusion into the study data set. The DCE-MRI data acquisition was designed to occur between the pre-contrast high-resolution acquisitions and the post-contrast high-resolution CE-MRI data acquisition required for RAMRIS scoring.

The protocol was based on a coronal 3D spoiled gradient echo acquisition (3D SPGR) on GE scanners, a 3D fast low angle shot (FLASH) or volume interpolated GRE (VIBE) acquisition on Siemens scanners and a 3D *T_1_*-fast field echo (T1-FFE) on Philips scanners; for brevity these will henceforth all be referred to as 3D gradient-recalled echoes (3D GRE). The protocol began with a coronal short-tau inversion recovery (STIR) scan and a fat-suppressed coronal *T_1_*-weighted 3D gradient echo (3D GRE) scan (“pre-contrast”). Prior to the dynamic series a baseline *T_1_* mapping acquisition was performed using three separate 3D GRE acquisitions with different flip angles. The dynamic series itself consisted of sequential 3D GRE acquisitions with a temporal resolution ranging between 9 s – 13 s, dependent on scanner performance characteristics. The total duration of the DCE-MRI series was limited to a maximum of 7 minutes in order to minimise the potential for patient discomfort and movement and to allow time for the other data acquisitions required for the study. Gd-CA was administered via a power injector (manual injection was not permitted) at 3 ml.s^-1^ at the start of the 6^th^ phase of the dynamic series. Sites were allowed to choose from a pre-defined list of eligible gadolinium-based contrast agents for the study: gadoterate (Dotarem), gadopentetate (e.g. Magnevist), gadobutrol (Gadovist), gadoversetamide (Optimark), gadoteridol (Prohance), or gadodiamide (Omniscan); each site was required to use the same agent throughout the study. The DCE-MRI series was followed by a final 3D GRE scan using the same parameters as those for the pre DCE-MRI 3D GRE scan. Supplementary table 3 shows typical key scanning parameters for the scanners used in this study.

**Supplementary table 3: Typical DCE-MRI acquisition parameters for each scanner manufacturer**

| ***Make*** | GE Healthcare | Siemens | Philips |
| --- | --- | --- | --- |
| ***Pulse Sequence*** | 3D FSPGR | 3D FLASH (‘fl_3d’) | 3D T1-FFE |
| ***Slice thickness [mm]*** | 1.5 | 1.5 | 1.5 |
| ***Acquisition matrix (frequency × phase)*** | 180 x 135 | 192 x 144 | 180 x 180 |
| ***Reconstruction matrix*** | 256 x 256 | 192 x 144 | 180 x 180 |
| ***Averages (NEX)*** | 0.5 | 1 | 1 |

Note: these parameters were optimised at each centre, depending on gradient performance, coil and MR scanner software version. All scans employed 3D gradient-recalled echoes and were performed in the coronal plane without fat suppression. Typical TE was 1.2 ms and typical TR was 4.8 ms. Flip angles of 2, 10, and 17 degrees were typically used for baseline *T_1_* measurement, and 17 degrees typically for the dynamic series post contrast. Field of view was 180 mm (readout) with 75% phase field of view. For baseline *T_1_* measurement, 16 dynamic repeats/phases were used, with 30-45 for the dynamic series post contrast

# Image Quality Control

During the live phase of the study, sites were required to repeat the phantom scanning (with temperature monitoring) at approximately 4-weekly intervals for the duration of their involvement in the study. Data were uploaded to the central analysis site to allow scanner performance to be monitored over time.

Correction for errors in *T_1_* due to variations in flip angle calibration between coils, scanners and over time was enabled by determining flip angle correction factors from the phantom data.

Failures in patient image QC during the initial image receipt process were due to technical, operator or subject-related errors. Technical QC failures included errors in flip angle setting and inconsistent radiofrequency transmitter/receiver gains resulting in unreliable image signal intensity and unreliable baseline *T_1_* measurements. Operator errors included protocol violations such as incorrect contrast agent flow rate, modification of image sequence parameters such as field of view, TR, TE. Subject-related errors were generally related to significant bulk motion, such as consistent movement of the hand/wrist during image acquisition and large in- and through-plane movement that could not be corrected by image registration methods. In these cases, repeat scans were requested.

During the analysis phase, images were failed if joints appeared to be too close to the edge of the field-of-view and susceptible to severe signal variations due to radiofrequency field in homogeneities. This generally affected the PIP joints, including IP-1.

In total, 122 data-sets provided DCE-MRI biomarkers of which 109 were included in the analyses in Table 1 and 2. Of these 20 were included despite minor QC violations, while 89 (82%) passed QC in full, and are noted as “high quality” in Supplementary figure 6.

# Region-of-Interest Definition

Three-dimensional statistical shape modelling[33] has previously shown advantages in the analysis of longitudinal studies using 3D musculoskeletal MR images[34,35]. From principal component analysis of a set of training examples, a model is constructed representing the anatomic average and anatomic variation in the training set. Regions of interest can be drawn on the anatomic average and automatically propagated back to each patient’s individual image at each time point, thereby avoiding segmenter bias. In this work, the ROIs were defined as joint voxel mask images that were generated for each of the joints being explored. To this end, 3D active appearance models (AAMs) were used to automatically identify bones, soft tissues and related anatomical regions of interest. The AAMs were built from an independent training set from a different cohort including 47 subjects with established RA, plus 20 subjects who did not have the disease. The training set was previously acquired using a Siemens VIBE (volumetric interpolated brain examination) acquisition sequence, which produces images which are very similar to those acquired in the present sub-study. In the training set, all wrist and hand bones (metacarpals, proximal and medial phalanges, hamate, capitate, trapezoid, trapezium, lunate, scaphoid, triquetrum, distal radius and distal ulna) were manually segmented, using EndPoint software (Imorphics, Manchester, UK). One AAM was built for each bone from the manual segmentations as previously described[34,36]. To model the soft tissues which formed a boundary edge around the capsules of the RAMRIS synovitis regions, the groupwise registration method was used as previously described[37,38] for knees. Soft tissues, including the synovial capsule, were added to the model by manual segmentation of the reference mean image generated during the groupwise model building process.

Two measures were provided: the ROI*, i.e.* the area in which enhancement post-Gd-CA is interpreted to reflect uptake by the synovium: broadly the capsular structure around each joint (or set of joints in the carpal bones) shown in Figure 1b and Supplementary Figure 3b; and the volume of enhancing pannus (*VEP*) *i.e.* voxels within the ROI which increased in intensity following administration of contrast shown in Supplementary Figure 4.

The workflow to identify the ROI was:

1. Identify the bones within each image by fitting an AAM to each bone in the pre-contrast and post-contrast images[36]. This was usually a fully automatic process but in a small number of cases where the hand was positioned unusually, or where disease had distorted the appearance, the search was initialised manually to increase the chances of success;
2. Identify the soft tissue around each synovial capsule by fitting the soft tissue AAMs to each pre-contrast image, initialised using the bones identified at step (a);
3. A 3D spline transformation was defined between the reference mean image and each pre-contrast image in the dataset using the search results of the bone AAMs and the soft tissue AAMs as constraints. This transformation was applied to the mean capsule surface, warping it to the pre-contrast image. This closed 3D surface was then converted into a binary voxel mask representing the ROI.

The workflow to identify the *VEP* was:

1. Pre- and post-contrast images were registered In order to compensate for patient movement. A 3D spline transformation was defined between the pre- and post-contrast images using the results of the bone AAM searches in each pair of images as constraints, and used to define a piecewise linear image registration.
2. *VEP* was defined as the volume within the ROI for each mask which enhanced following administration of Gd-CA. *VEP* was identified by creating a subtraction image from the pre- and post-contrast images, using a shuffle transform[39]. The subtraction image was thresholded to remove background noise, discarding values less than the 90^th^ percentile.

# DCE-MRI time series analysis and compartmental modelling

All DCE-MRI data were transferred to the central analysis site (Bioxydyn Limited, Manchester, UK) where they were transformed into contrast agent concentration units with the aid of *T_1_* maps calculated from the baseline 3D GRE acquisitions (Supplementary table 3), with flip angles corrected using the phantom-derived calibration factors on a site-by-site basis. All DCE-MRI data were processed using tracer kinetic model-based analysis and heuristic model-free analyses. The extended Tofts model[40] was applied to the data with a population arterial input function[27], corrected for patient haematocrit at each scanning visit[28]. The extended Tofts model allowed estimates of the volume transfer coefficient of the contrast agent *K*^trans^, the leakage volume *v_e_* and the blood plasma volume *v_p_* to be extracted voxelwise within the regions of interest.

Four heuristic, model-free parameters were also calculated. The initial area under the concentration time course was calculated[41] over 60 s and 120 s (*IAUC*_60_ and *IAUC*_120_). The initial rate of enhancement (*IRE*) and maximum enhancement (*ME*)[42] were calculated with the aid of the model fits determined using the extended Tofts model: the fitted time course of the model (in concentration units) allowed low-noise estimates of both parameters to be extracted voxelwise. This approach also allowed consistent definition of the timing for definition of IRE, as the continuous model fit-derived concentrations naturally allow for interpolation between the time points of data acquisition, which varied slightly between centres. Figures 1 and Supplementary figure 3 show example DCE-MRI parameterisations obtained from the joint voxel mask images.

All parameters were calculated as mean values over the two regions defined above for each joint – the whole joint ROI and the region defined as *VEP*. Supplementary figure 4 shows example 3D rendered wrist and hand bones with areas of synovitis overlaid. The *VEP* values submitted for statistical analysis were each the sum of individual joints’ *VEP*. For joints where *VEP* was not analysable it was set to 0. For each DCE-MRI parameter, a voxelwise mean value for each joint was calculated with non-*VEP* voxels set to zero, and with any voxels which failed to provide a fit to the extended Tofts model discarded. Also voxels were rejected for all parameters when the baseline *T*_1_ model fit failed (most rejected voxels were due to fit failures at the *T*_1_ fitting stage). The values submitted for statistical analysis were the jointwise means of each joint’s respective voxelwise means. The decision to average some of the biomarkers jointwise (i.e. equal weighting to all joints whether diseased or not), rather than voxelwise over the entire hand’s inflamed synovium, may have adversely affected the reported repeatability, particularly as the severity of joint disease was lower than anticipated, and therefore level of enhancement seen by the majority of joints was too low to obtain reliable DCE-MRI readouts.

The time series of enhancement observed for most joints was an extended increase in signal intensity that did not reach a maximum over the duration of the DCE-MRI session (Supplementary figure 5). Under these conditions *v_e_* is ill- defined and sensitive to noise, with a wide range of possible values consistent with the time series. The presence of this prolonged signal intensity increase may be caused by the presence of convection or diffusion of contrast agent away from the supplying capillary beds where leakage occurs, a possibility that is likely to lead to unphysical estimates of leakage volume. *v_e_* values were therefore not further evaluated for this study.

The acquisition protocol for this study imaged the whole hand within a knee coil, and attempted to include the first PIP joint for the thumb and fingers along with the RAMRIS regions. This was not successful, and the majority of the PIP joints were rejected during the quality review process. Around one-third of the images did not include the complete region of interest for all PIP joints. In the remainder, image quality was poor, either due to low signal to noise (in the finger PIPs), or fat-saturation failure (in the thumb PIP). As a consequence, these joints were excluded from DCE analysis.

The attempt to image the PIP joints also had an adverse effect on image quality in the radio-ulnar region. For over half of the images in the dataset the quality of the ROI for this RAMRIS region was inadequate, or partially absent, for the DCE-MRI and VEP analyses, and according to the protocol these results were returned as ‘No Value Recorded’. However, in virtually all patients the radiologists were nonetheless able to determine a RAMRIS synovitis score for this region. Comparison of changes in VEP and DCE values with RAMRIS values should take account of these missing data.

# RAMRIS scoring

OMERACT RAMRIS scoring was performed by two independent radiologists experienced in the method. All serially acquired images of an individual patient were presented to the radiologists simultaneously but in random order, with the acquisition dates masked, so that the radiologists were blinded to time-order. RAMRIS synovitis score is the sum of the scores assigned by an experienced musculoskeletal radiologist to three wrist regions (distal-radioulnar, radiocarpal, intercarpal-CMC) and the five metacarpalphalangeal joints, where each region or joint is scored from 0 to 3 (0 = Normal/No Synovitis; 1 = Synovitis involving 1% to 33% of the estimated maximally distended synovial cavity; 2= Synovitis involving 34% to 67% of the estimated maximally distended synovial cavity; 3= Synovitis involving 68% to 100% of the estimated maximally distended synovial cavity). The maximum RAMRIS synovitis score achievable was therefore 24. Scores from the two radiologists were averaged.

# Statistical analysis

Statistical analyses were performed using SAS® Version 8.0 or higher and, where appropriate, additional validated software. Analyses were performed in accordance with a predetermined Statistical Analysis Plan, finalised and signed prior to locking the database and unblinding. For all efficacy analyses fostamatinib was compared pairwise to each of placebo (at week 6) and adalimumab (at week 6 and week 24). Efficacy endpoints were tested at a 2-sided significance level of 10% for fostamatinib versus placebo, and for fostamatinib versus adalimumab. Since this is a Phase II trial, no adjustments were made for multiplicity. No imputation of missing MRI data was carried out. In particular data were not imputed for those patients who discontinued the study due to its early termination. All DCE-MRI endpoints were log-transformed prior to analysis. The log of the ratio in each contrast enhanced MRI endpoint (*K^trans^, v_e_, v_p_, IRE, IAUC_60_, IAUC_120_, VEP and ME*) at week 6 and week 24 respectively over baseline, was analyzed using an analysis of covariance (ANCOVA). An ANCOVA model of the log ratio to baseline was fitted including terms for log transformed baseline as a continuous covariate and treatment and DMARD naïvety (DMARD naïve *vs*. DMARD-IR/intolerant) as fixed factors. Results were presented in terms of the adjusted means for each treatment group, and estimates of treatment difference back-transformed so as to be presented as a ratio with associated 2-sided 90% confidence interval (CI) and p-value for each treatment comparison.

In addition, for those patients for whom two baseline DCE-MRI scans were completed, data were used to assess the reproducibility of the DCE-MRI biomarkers via the use of a mixed model of the two baseline results only. Given that only baseline results were utilised, all such patients with two baseline assessments from any treatment group were included, with no fixed factor for treatment. An Analysis of Variance (ANOVA) model was fitted to each log-transformed DCE-MRI parameter, including subject as a random effect, and the inter-subject and intra-subject components of variation were obtained and presented as a standard deviation on the log scale together with the coefficient of variation (CoV).

The analysis of the change in OMERACT RAMRIS synovitis score was performed pairwise at week 6 (fostamatinib in comparison to placebo, and fostamatinib in comparison to adalimumab) and at week 24 (fostamatinib in comparison to adalimumab) using a van Elteren test, with DMARD naïvety as a stratification variable. The unstratified Hodges Lehmann non-parametric point estimate for the median difference between treatment groups was also presented with its associated 90% CI.

The standardized response mean (SRM) was calculated from the response at 6 weeks in the adalimumab group. This is provided at the suggestion of one of the reviewers, as a convenience to the reader. However it should be noted that the DCE-MRI parameters were known to have skewed distributions meaning that an approach using the mean and standard deviation based upon a normality assumption was not appropriate. To be valid an SRM would rely on this assumption.

##

##


**Figure 6 (supplementary material)**Patient Disposition. For the 25 patients who withdrew after baseline, the main reason (N=17) was that the study stopped early

# Supplementary References

1. Østergaard M, Peterfy C, Conaghan P, *et al.* OMERACT Rheumatoid Arthritis Magnetic Resonance Imaging Studies. Core set of MRI acquisitions, joint pathology definitions, and the OMERACT RA-MRI scoring system. *J Rheumatolology*  2003;6:1385-6.

2. Haavardsholm EA, Østergaard M, Ejbjerg BJ, *et al.* Reliability and sensitivity to change of the OMERACT rheumatoid arthritis magnetic resonance imaging score in a multireader, longitudinal setting. *Arthritis & Rheumatology* 2005;52:3860-7.

3. Peterfy C, Østergaard M, Conaghan PG. MRI comes of age in RA clinical trials. *Annals of the Rheumatic Diseases* 2013;72:794-6.

4. Kessler LG, Barnhart HX, Buckler AJ, *et al.* The emerging science of quantitative imaging biomarkers terminology and definitions for scientific studies and regulatory submissions. *Statistical Methods in Medical Research* 2015;24:9-26

5. Tofts PS, Brix G, Buckley DL, *et al.* Estimating kinetic parameters from dynamic contrast-enhanced *T_1_* weighted MRI of a diffusable tracer: standardized quantities and symbols. *Journal of Magnetic Resonance Imaging* 1999;10:223-32.

6. O'Connor JPB, Jackson A, Parker GJM, *et al.* Dynamic contrast-enhanced MRI in clinical trials of antivascular therapies. *Nature Reviews Clinical Oncology* 2012;9:167-77.

7. Hodgson RJ, O'Connor P, Moots R. MRI of rheumatoid arthritis image quantitation for the assessment of disease activity, progression and response to therapy. *Rheumatology (Oxford)* 2008;47:13-21.

8. Beals C, Baumgartner R, Peterfy C, *et al.* Treatment effects measured by dynamic contrast enhanced MRI and RAMRIS for rheumatoid arthritis. *Annals of the Rheumatic Diseases* 2013;72:Suppl 3 A748

9. Reece RJ, Kraan MC, Radjenovic A, *et al.* Comparative assessment of leflunomide and methotrexate for the treatment of rheumatoid arthritis, by dynamic enhanced magnetic resonance imaging. *Arthritis & Rheumatology* 2002;46:366-72.

10. Taylor PC, Genovese MC, Greenwood M, *et al*. OSKIRA-4: a phase IIb randomised, placebo-controlled study of the efficacy and safety of fostamatinib monotherapy. *Annals of the Rheumatic Diseases* 2015;74:2123-2129

11. Genovese MC, Kavanaugh A, Weinblatt ME, *et al.* An oral Syk kinase inhibitor in the treatment of rheumatoid arthritis: a three-month randomized, placebo-controlled, phase II study in patients with active rheumatoid arthritis that did not respond to biologic agents. *Arthritis & Rheumatology* 2011;63:337-45.

12. Waterton JC, Rajanayagam V, Ross BD, *et al.* Magnetic resonance methods for measurement of disease progression in rheumatoid arthritis. *Magnetic Resonance Imaging* 1993;11:1033-8.

13. Waterton JC. Translational Magnetic Resonance Imaging and Spectroscopy: Opportunities and Challenges. *In* Garrido, L and Beckmann, N (Eds), New Applications of NMR in Drug Discovery and Development. Cambridge UK: RSC press. 2013: 333-60.

14. Hodgson RJ, Connolly S, Barnes T, *et al.* Pharmacokinetic modeling of dynamic contrast-enhanced MRI of the hand and wrist in rheumatoid arthritis and the response to anti-tumor necrosis factor-alpha therapy. *Magnetic Resonance in Medicine* 2007;58:482-9.

15. United States Department of Health and Human Services, Food and Drug Administration. Guidance for Industry - Rheumatoid Arthritis:Developing Drug Products for Treatment (DRAFT GUIDANCE Revision 1). 2011.

16. European Medicines Agency. Draft guideline on clinical investigation of medicinal products other than non-steroidal anti-inflammatory drugs for treatment of rheumatoid arthritis. 2011;CPMP/EWP/556/95 Rev. 2.

17. Gandjbakhch F, Conaghan PG, Ejbjerg B, Haavardsholm EA, Foltz V, Brown AK, *et al.* Synovitis and osteitis are very frequent in rheumatoid arthritis clinical remission: results from an MRI study of 294 patients in clinical remission or low disease activity state. *Journal of Rheumatology* 2011;38:2039-2044.

18. McQueen F, Naredo E. The 'disconnect' between synovitis and erosion in rheumatoid arthritis: a result of treatment or intrinsic to the disease process itself? *Annals of the Rheumatic Diseases* 2011;70:241-244.

19. Conaghan PG, Peterfy C, Olech E, Kaine J, Ridley D, Dicarlo J, *et al.* The effects of tocilizumab on osteitis, synovitis and erosion progression in rheumatoid arthritis: results from the ACT-RAY MRI substudy. *Annals of the Rheumatic Diseases* 2014;73:810-6

20. Dohn UM, Ejbjerg B, Boonen A, Hetland ML, Hansen MS, Knudsen LS, *et al*. No overall progression and occasional repair of erosions despite persistent inflammation in adalimumab-treated rheumatoid arthritis patients: results from a longitudinal comparative MRI, ultrasonography, CT and radiography study. *Annals of the Rheumatic Diseases* 2011;70:252-258.

21. Peterfy C, Durez P, Haraoui B, Sinisi S, Meerwein S, Kupper H. RESPONSE OF EARLY RHEUMATOID ARTHRITIS (RA) TO TREATMENT WITH ADALIMUMAB PLUS METHOTREXATE VS. METHOTREXATE ALONE: MAGNETIC RESONANCE IMAGING RESULTS FROM OPTIMA. *Annals of the Rheumatic Diseases* 2010;69(Suppl3):455.

22. Fritz J, Galeczko EK, Schwenzer N, Fenchel M, Claussen CD, Carrino JA, *et al*. Longitudinal changes in rheumatoid arthritis after rituximab administration assessed by quantitative and dynamic contrast-enhanced 3-T MR imaging: preliminary findings. *European Radiology* 2009;19:2217-2224.

23. Buch MH, Boyle DL, Rosengren S, Saleem B, Reece RJ, Rhodes LA, *et al.* Mode of action of abatacept in rheumatoid arthritis patients having failed tumour necrosis factor blockade: a histological, gene expression and dynamic magnetic resonance imaging pilot study. *Annals of the Rheumatic Diseases* 2009;68:1220-1227.

24. Tam LS, Griffith JF, Yu AB, Li TK, Li EK. Rapid improvement in rheumatoid arthritis patients on combination of methotrexate and infliximab: clinical and magnetic resonance imaging evaluation. *Clinical Rheumatology* 2007;26:941-946.

25. Ostergaard M, Stoltenberg M, Henriksen O, Lorenzen I. Quantitative assessment of synovial inflammation by dynamic gadolinium-enhanced magnetic resonance imaging. A study of the effect of intra-articular methylprednisolone on the rate of early synovial enhancement. *British Journal of Rheumatology* 1996;35:50-59.

26. Hodgson RJ, Barnes T, Connolly S, Eyes B, Campbell RS, Moots R. Changes underlying the dynamic contrast-enhanced MRI response to treatment in rheumatoid arthritis. *Skeletal Radiology* 2008;37:201-207.

27. Parker GJ, Roberts C, Macdonald A, Buonaccorsi GA, Cheung S, Buckley DL, *et al.* Experimentally-derived functional form for a population-averaged high-temporal-resolution arterial input function for dynamic contrast-enhanced MRI. *Magnetic Resonance in Medicine* 2006;56:993-1000.

28. Roberts C, Hughes S, Naish JH, Holliday K, Watson Y, Cheung S, *et al.* Use of An Individually Measured Hematocrit in DCE-MRI studies. *Proc. Int. Soc. Magn. Reson. Med*. 2011:2221.

29. Roberts C, Issa B, Stone A, Jackson A, Waterton JC, Parker GJM. Comparative study into the robustness of compartmental modeling and model-free analysis in DCE-MRI studies. *Journal of Magnetic Resonance Imaging* 2006;23:554-563.

30. Leach MO, Morgan B, Tofts PS, Buckley DL, Huang W, Horsfield MA, *et al.* Imaging vascular function for early stage clinical trials using dynamic contrast-enhanced magnetic resonance imaging. *European Radiology* 2012;22:1451-1464.

31. Lerski RA, de Certaines JD. Performance assessment and quality control in MRI by Eurospin test objects and protocols. *Magnetic Resonance Imaging* 1993;11:817-833.

32. Peterfy CG, Olech E, Dicarlo JC, Merrill JT, Countryman PJ, Gaylis NB. Monitoring cartilage loss in the hands and wrists in rheumatoid arthritis with magnetic resonance imaging in a multi-center clinical trial: IMPRESS (NCT00425932). *Arthritis Research & Therapy* 2013;15:R44.

33. Davies RH, Twining C, Taylor CJ. Statistical Models of Shape: Optimisation and Evaluation. Heidelberg, Germany: Springer; 2008.

34. Hunter DJ, Bowes MA, Eaton CB, Holmes AP, Mann H, Kwoh CK, et al. Can cartilage loss be detected in knee osteoarthritis (OA) patients with 3-6 months' observation using advanced image analysis of 3T MRI?. *Osteoarthritis and Cartilage* 2010;18:677-683.

35. Williams TG, Holmes AP, Waterton JC, Maciewicz RA, Hutchinson CE, Moots RJ, *et al.* Anatomically corresponded regional analysis of cartilage in asymptomatic and osteoarthritic knees by statistical shape modelling of the bone. *IEEE Transactions on Medical Imaging* 2010;29:1541-1559.

36. Cootes,T.F., Edwards,G.J., Taylor CJ. Active appearance models. *IEEE Trans. Pattern Anal. Mach. Intell.* 2001;23:681-685.

37. Williams TG, Vincent GR, Cootes TF, Balamoody S, Hutchinson CE, Waterton JC, *et al.* Automatic segmentation of bones and inter-image anatomical correspondence by volumetric statistical modelling of knee MRI. Biomedical Imaging: *From Nano to Macro, 2010 IEEE International Symposium on,* 2010:432-435.

38. Cootes TF, Twining CJ, Petrovic VS, Schestowitz RS, Taylor CJ. Groupwise Construction of Appearance Models using Piece-wise Affine Deformations. *In:* Clocksin WF, Fitzgibbon AW, Torr PHS, editors. *Proceedings of the British Machine Conference*: BMVA Press; 2005. p. 88.1-88.10.

39. Xanthopoulos E, Hutchinson CE, Adams JE, Bruce IN, Nash AFP, Holmes AP, *et al.* Improved wrist pannus volume measurement from contrast-enhanced MRI in rheumatoid arthritis using shuffle transform. *Magnetic Resonance Imaging* 2007;25:110-116.

40. Tofts PS. Modeling tracer kinetics in dynamic Gd-DTPA MR imaging. *Journal of Magnetic Resonance Imaging* 1997;7:91-101.

41. Evelhoch JL. Key factors in the acquisition of contrast kinetic data for oncology. *Journal of Magnetic Resonance Imaging* 1999;10:254-259.

42. Ostergaard M, Stoltenberg M, Lovgreen-Nielsen P, Volck B, Sonne-Holm S, Lorenzen I. Quantification of synovistis by MRI: correlation between dynamic and static gadolinium-enhanced magnetic resonance imaging and microscopic and macroscopic signs of synovial inflammation. *Magnetic Resonance Imaging* 1998;16:743-754.
